# Bridging Integrator 3 (BIN3) Downregulation Predicts a Poor Prognosis in Patients with Esophagus Carcinoma: A Study based on TCGA Data

**DOI:** 10.2174/1386207326666221205101815

**Published:** 2023-06-12

**Authors:** Daohang Li, Weiming Deng, Guozheng Huang, Xin Xiao

**Affiliations:** 1 Department of Thoracic Surgery, Chaohu Hospital Affiliated with Anhui Medical University, Hefei, 238000, China

**Keywords:** Esophagus carcinoma (ESCA), bridging integrator 3 (BIN3), prognosis, immune infiltration, TCGA, epithelial-mesenchymal transition (EMT)

## Abstract

**Background:**

Bridging integrator 3 (BIN3) has been reported to play a key role in certain tumors. Nevertheless, little is known about the role and clinical value of BIN3 in esophagus carcinoma (ESCA). This study aimed to investigate the pathological and prognostic role of BIN3 in ESCA patients.

**Methods:**

Genes significantly correlated with the prognosis of ESCA patients were screened and identified by comprehensive analysis of differentially expressed genes associated with overall survival (OS), disease-specific survival (DSS) and progression-free interval (PFI) in ESCA. The expression of BIN3, pathological features correlation and subgroup overall survival analysis were performed using The Cancer Genome Atlas (TCGA) and GTEx databases. Moreover, the potential signaling pathways in which BIN3 was involved were analyzed by GO-KEGG enrichment analysis and gene set enrichment analysis (GSEA). Immune infiltrates correlation of BIN3 in ESCA was performed by TIMER and ssGSEA. The influence of BIN3 on epithelial-mesenchymal transition (EMT) was validated by western blot.

**Results:**

There were two differentially expressed genes related to the prognosis of ESCA patients, which were identified from three gene clusters associated with overall survival (OS), disease-specific survival (DSS) and progression-free interval (PFI) in ESCA patients. The BIN3 mRNA level was found to be significantly decreased in ESCA compared to normal tissues (*p <* 0.05). The decreased expression of BIN3 in ESCA was significantly correlated with the clinical stage (p = 0.015), T stage (*p <* 0.05), histological type (*p <* 0.001), age (*p <* 0.05) and gender (*p <* 0.05). ESCA patients with high BIN3 expression were observed to be correlated with T stage (T3 & T4), age (<=60), gender (male), primary therapy outcome (PD) and columnar metaplasia (No) of favorable OS. GO-KEGG enrichment analysis revealed that BIN3 was involved in endocytosis. GSEA showed that several pathways were enriched in BIN3, such as O linked glycosylation of mucins, PID HNF3B pathway, biocarta TFF pathway, WP pregnane X receptor pathway, reactome regulation of beta cell development, WP Urea cycle and associated pathways and others. BIN3 was significantly related to the infiltration level of T cells (*p <* 0.001), Tregs (*p <* 0.001), B cells (*p <* 0.001), NK cells (*p <* 0.001), and macrophage M2 (*p <* 0.001). In addition, BIN3 overexpression inhibited N-cadherin expression and promoted E-cadherin expression in ESCA cell lines TE-1.

**Conclusion:**

These results suggest that BIN3 might be a potential prognostic biomarker in ESCA. BIN3 functions as a tumor-suppressor role in ESCA, which is significantly associated with the immune infiltration of ESCA.

## 1 INTRODUCTION

Esophagus carcinoma (ESCA) is one of the most common malignancies in the world and the fourth leading cause of cancer-related death in China. There are two major histological subtypes including esophagus squamous cell carcinoma (ESCC) and esophageal adenocarcinoma (EAC) [1, 2]. In recent years, the improvements have been made in the diagnosis and treatment of ESCA patients, however, the clinical outcome of ESCA patients was not optimistic. Studies have shown that recurrence and metastasis are the main causes of poor prognosis [3, 4]. Thus, it is essential to investigate more effective biomarkers for prognosis prediction and evaluation for ESCA patients.

Nowadays, the therapeutic strategies for ESCA have been emerging multimodality approaches [3, 5] including surgical techniques, chemotherapy, targeted therapy and immunotherapy. Due to the advances in treatment, the clinical outcomes have been improved in ESCA patients at earlier stages. However, the general outcome is still unfavorable. It is urgent and necessary to investigate susceptible genes to improve ESCA patient survival rates.

Bridging integrator 3 (BIN3) is a ubiquitously expressed member of the BAR domain protein family which has been reported to participate in endocytosis, cell motility, and some other biological processes [6]. A study showed that loss of BIN3 led to cataracts and increased susceptibility to lymphoma during aging in the mouse model [7]. It is studied that BIN3 could form a complex with Rac1 and Cdc42 to regulate myogenesis in skeletal muscle [8]. It is worth mentioning that BIN3 was located at human chromosome 8p21.3, the loss of which region could cause non-Hodgkin’s lymphoma and some epithelial tumors [9, 10]. It was observed that symptoms associated with the development of lung cancer occurred when BIN3-deletion mice were treated with carcinogens. These studies suggest that BIN3 might play a critical role in tumor inhibition.

Up to now, the role of BIN3 in various tumors especially in ESCA is still unclear. In this study, we investigated the expression and clinical significance of BIN3 in ESCA via TCGA and GTEx databases. Firstly, we verified the key prognostic factors in ESCA tumors samples by screening three different gene sets associated with prognosis. Secondly, we analyzed the expression, clinical features and prognostic values of BIN3 in ESCA using various packages of R software. Moreover, the relationship between immune cells and BIN3 expression was also assessed. Our work first reveals that BIN3 may serve as a prognostic biomarker for ESCA.

## 2 METHODS

### 2.1 Data Analysis

The mRNA expression profiles, clinicopathological data and clinical outcomes of ESCA human samples were downloaded from TCGA (https://portal.gdc.cancer.gov/) and GTEx (https://gtexportal.org/) databases [11]. The expression data of BIN3 in ESCA was also obtained from the GEPIA2 database (GEPIA 2 (cancer-pku.cn)) [12].

### 2.2 Prognosis Analysis

The overall survival (OS), Disease-specific survival (DSS) and Progression-free interval (PFI) associated genes in ESCA were analyzed using survival [v3.2-10] and survminer [v0.4.^9^] R package [11]. The overlaps of the above three genesets were analyzed and visualized using ggplot2 [v3.3.^3^].

### 2.3 GO and KEGG Enrichment Analysis

The binding proteins with BIN3 were analyzed using STRING: functional protein association networks (string-db.org) [13]. The top 100 related genes with BIN3 were obtained from the GEPIA2 database [12]. The gene ontology (GO) and Kyoto Encyclopedia of Genes and Genomes (KEGG) analysis was performed using ClusterProfiler [v3.14.^3^] R package [14].

### 2.4 Gene Set Enrichment Analysis (GSEA)

The differential genes with BIN3 in ESCA were analyzed using DESeq2 [v1.26.0] [15]. The GSEA analysis and visualization were performed using clusterProfiler [v3.14.^3^] [14, 16]. The reference gene set was c2.cp.v7.2.symbols.gmt (Curated). It was considered to be significant on the statistics that the false discovery rate (FDR) < 0.25 and the adjusted *p* (p.adjust) < 0.05.

### 2.5 Immune Infiltration Analysis

The twenty-four immune cells were obtained from the research of Bindea *et al*. [17]. The correlation analysis between BIN3 and these immune cells was performed using GSVA [v1.34.0] within the ssGSEA algorithm [18]. The immune infiltration levels of BIN3 in the tumor environment were conducted by the TIMER2.0 (cistrome.org) [19].

### 2.6 Quantitative Real-time Polymerase Chain Reaction (qRT-PCR)

The total RNA from cells was extracted using TRIzol reagent (Thermo Fisher Scientific, MA, USA) and reverse-transcribed into cDNA with PrimeScript RT Master Mix (Takara, Dalian, China). Quantitative RT-PCR was performed on an Applied Biosystems™ 7500 (Applied Biosystems). The human GAPDH was used as the control. The primers were listed as follows. BIN3, F: 5’- GGAAAACT TCAGCACCATGTCA-3’;R: 5’-CTCACTG AGTTTGGCCTCGT-3’. GAPDH, F: 5’- GAGAAGGCTGGGGC TCATTT-3’;R: 5’-AGTGATGGCATGGACTGTGG-3’.

### 2.7 Western Blot

Normal human esophagus epithelial cell line Het-1A (IM-H274, Immocell Biotechnology Co.,Ltd., Xiamen, China) and human ESCA cell line TE-1 (CL-0231, Procell Science&Technology Co.,Ltd., Wuhan, China), KYSE30 (CL-0577, Procell), KYSE-150 (CL-0638, Procell) were cultured in RPMI-1640 medium (Gibco, Carlsbad, CA, USA) with 10% fetal bovine serum (FBS, Gibco, USA), supplemented with 100 U/mL streptomycin and penicillin. Cells were lysed with RIPA buffer (Cell Signaling Technology, Danvers, MA, USA). The equal amounts of proteins were separated by sodium dodecyl sulfate-polyacrylamide gel electrophoresis (SDS-PAGE) and transferred onto polyvinylidene difluoride (PVDF) membranes (Millipore, Bedford, MA, USA). The membranes were blocked with 5% non-fat milk for one hour at room temperature. The membranes were incubated with antibody against BIN3 (TA502145, 1:2000, OriGene Technologies, Wuxi, China), E-cadherin (ab1416, 1:1000, Abcam, Cambridge, UK), N-cadherin (ab76011, 1:5000, Abcam) overnight at 4°C. The membranes were incubated with corresponding secondary antibodies.

### 2.8 Statistical Analysis

All data analyses were conducted by R version 3.6.3. The expression difference of BIN3 in ESCA tumor and normal tissues was analyzed using the Wilcoxon rank sum test. The correlation between BIN3 expression and the clinical features was analyzed using logistic regression, Wilcoxon rank sum test, Fisher exact test, or chi-squared test. Prognosis analysis including overall survival, disease specific survival and progress free interval was performed using Kaplan-Meier method and Cox regression. Subgroup overall survival analysis was performed using Kaplan-Meier method and Cox regression. *p <* 0.05 was considered statistically significant.

## 3 RESULTS

### 3.1 Screening of Prognosis-Related Genes in ESCA

We screened a total of 87 genes in ESCA associated with overall survival (OS, *p <* 0.01, Supplemental Table **1**). Using the same method, we obtained a total of 78 genes in ESCA correlated with disease-specific survival prognosis (DSS, *p <* 0.01, Supplemental Table **2**) and 71 genes with progression-free interval prognosis (PFI, *p <* 0.01, Supplemental Table **3**), respectively. The intersection was displayed in Fig. (**1A**). There were only two genes obtained including BIN3 and AXIN1 across the above-mentioned three clusters. The OS, DSS, PFI curves of these two genes were shown in Figs. (**1B**-**G**). These results suggest that high expression of BIN3 or AXIN1 predicts a favorable prognosis in ESCA patients, playing a tumor suppressor role in ESCA. However, the expression analysis showed that AXIN1 was highly expressed in ESCA tumor tissues compared to adjacent normal tissues (Supplementary Fig. **1**), contradicting the prognosis results. Thus, BIN3 was selected for the subsequent research.

### 3.2 Expression and Clinical Correlation of BIN3 in ESCA

**Fig. (2):**
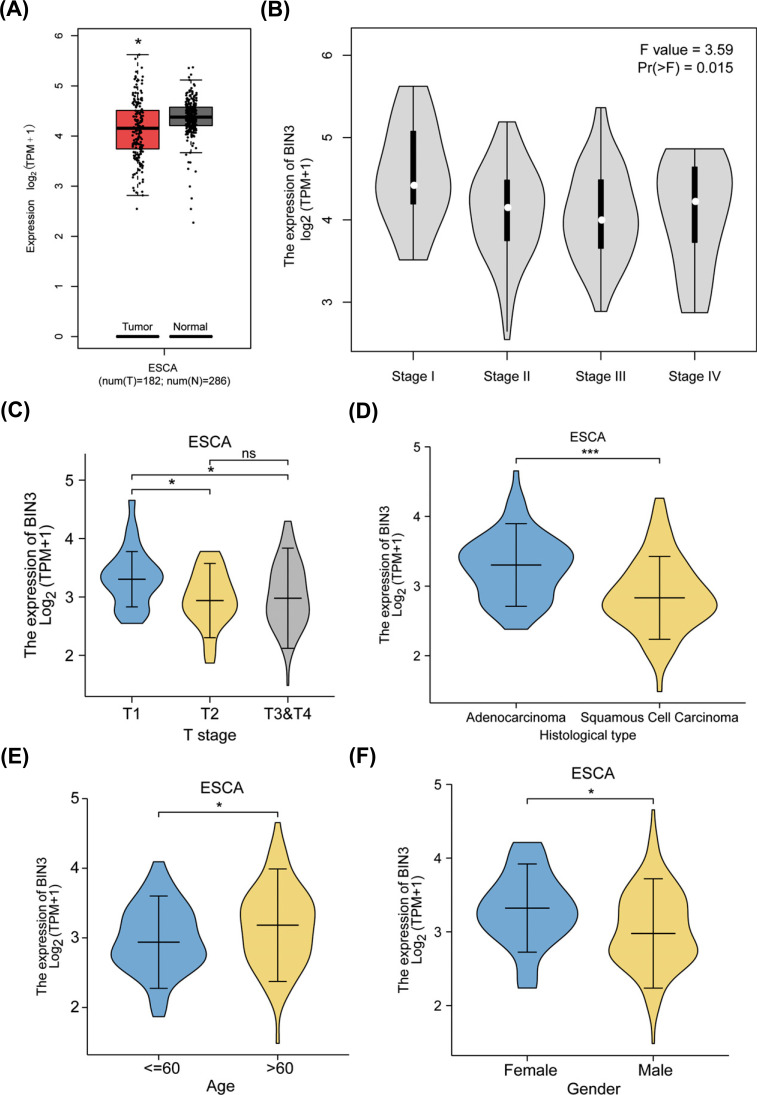
Clinical features with BIN3 mRNA in ESCA. (**A**) The mRNA of BIN3 was analyzed in ESCA using GEPIA2 (*p <* 0.05). (**B**) The clinical stages with BIN3 expression in ESCA were analyzed (p = 0.015). (**C**) The correlation analysis between T stages and BIN3 expression was performed in TCGA database (*p <* 0.05). (**D**) BIN3 expression was assessed in adenocarcinoma and squamous cell carcinoma of ESCA (*p <* 0.001). (**E**) The correlation analysis between age and BIN3 expression in ESCA was performed (*p <* 0.05) (**F**) The correlation analysis between gender and BIN3 expression in ESCA was performed (*p <* 0.05).

BIN3 expression was observed to be decreased in ESCA tumor tissues compared to normal tissues from TCGA and GTEx database (Fig. **2A**). The level of BIN3 decreased significantly in different advanced stages of ESCA (Fig. **2B**, *p* =0.015). What’s more, clinical correlation analysis showed there was a significant association between BIN3 expression and T stage (Fig. **2C**, *p <* 0.05), histological type (Fig. **2D**, *p <* 0.001), age (Fig. **2E**, *p <* 0.05) and gender (Fig. **2F**, *p <* 0.05). Consistently, these findings were in accordance with the analysis as shown in Table **1** using the Fisher test or chi-square test. What’s more, logistic regression analysis (Table **2**) showed that there was a strong correlation between BIN3 expression and certain clinical features, such as N stage (N1&N2&N3 *vs*. N0) (Odds Ratio(OR) = 0.424, 95% confidence interval (CI): 0.215-0.825, *p* = 0.012), Pathologic stage (OR = 0.462, 95% CI: 0.230-0.910, *p* = 0.027), Tumor cental location (OR = 0.420, 95% CI: 0.205-0.836, *p* = 0.015), and Columnar mucosa dysplasia (OR = 5.556, 95% CI: 1.859-19.277, *p* = 0.004), but not T stage (OR = 0.654, 95% CI: 0.336-1.262, *p* = 0.207), M stage (OR = 1.810, 95% CI: 0.425-9.141, *p* = 0.430), and Columnar metaplasia (OR = 1.545, 95% CI: 0.639-3.851, *p* = 0.339). The above results revealed that BIN3 is involved in ESCA development and progression.

### 3.3 Subgroup Overall Survival Analysis of BIN3 in ESCA

Next, we investigated the relationship between BIN3 expression and the overall survival of ESCA patients with different clinical characteristics as shown in Fig. (**3**). The Kaplan-Meier curves showed that high expression of BIN3 was associated with better overall survival of ESCA patients at T stage (T3&T4), Age < =60, Gender (male), primary therapy outcome: PD and Columnar metaplasia: No (all *p <* 0:05). However, the univariate analysis (Table **3**) revealed that only M stage and pathologic stage had a correlation with the prognosis of ESCA patients with BIN3 expression (all *p <* 0.001). Furthermore, the multivariate analysis verified that M stage, pathologic stage, and BIN3 expression could be considered prognostic favorable factors of OS (HR = 2.831 (1.362-5.883), *p* = 0.005, Table **3**) in patients with ESCA. These data further suggest that BIN3 is an independent prognostic biomarker for ESCA patients.

### 3.4 Enrichment Analysis of BIN3-related Partners

**Fig. (4):**
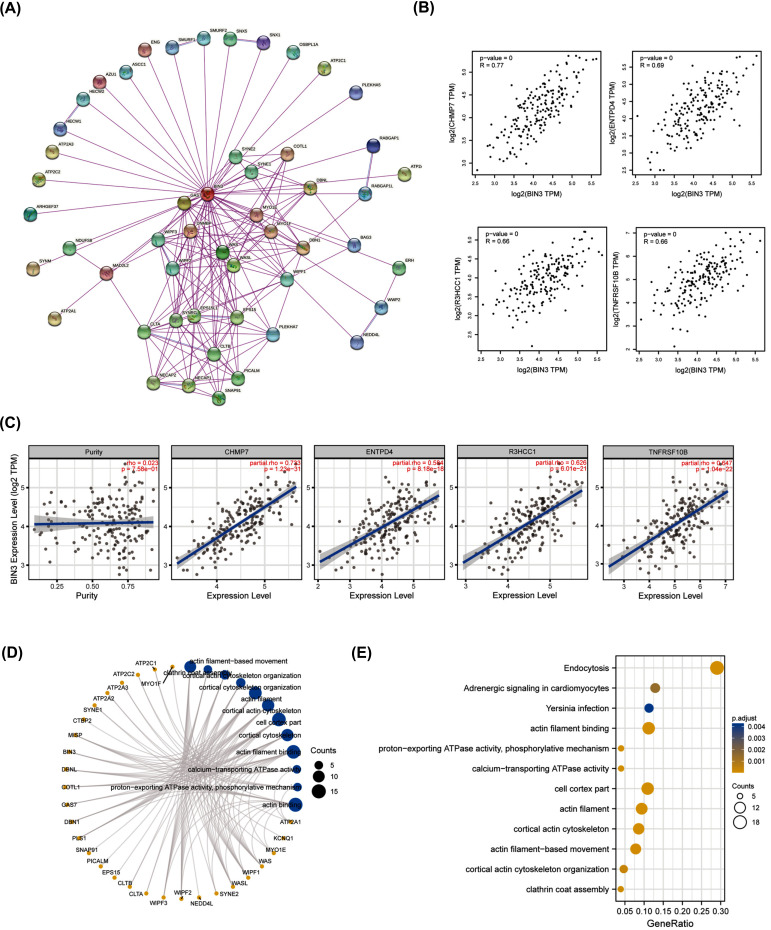
Enrichment analysis of BIN3-related genes. (**A**) The binding proteins with BIN3 were analyzed using STRING online tool. (**B**) The top four correlated genes with BIN3 were displayed by GEPIA2, including CHMP7, ENTPD4, R3HCC1, and TNFRSF10B. (**C**) The correlation between BIN3 and CHMP7, ENTPD4, R3HCC1, and TNFRSF10B was verified by TIMER2 database. (**D**) GO analysis was displayed by the visual network analysis with the BIN3-binding and correlated genes. (**E**) GO and KEGG pathway analysis was conducted with the BIN3-binding and correlated genes.

Furthermore, we explored the regulatory mechanism of BIN3-involved in ESCA tumorigenesis. Firstly, we obtained fifty BIN3-binding proteins via STRING, which were proved by experimental evidence (Fig. **4A**). Next, we screened a total of the top 100 genes related with BIN3 expression by GEPIA2 database. Fig. (**4B**) showed that there was a positive relationship between BIN3 and CHMP7 (charged multivesicular body protein 7, R = 0.77), ENTPD4 (ectonucleoside triphosphate diphosphohydrolase 4, R = 0.69), R3HCC1 (R3H domain and coiled-coil containing 1, R = 0.66) and TNFRSF10B (TNF receptor superfamily member 10b, R = 0.66) genes (all *p <* 0.001). We also validated the relationship between BIN3 and the above 4 genes in ESCA *via* TIMER2 online tool (Fig. **4C**). We performed GO and KEGG enrichment analyses by combining BIN3-binding proteins and correlated genes. We combined the two datasets to perform KEGG and GO enrichment analyses. The GO enrichment analysis revealed that BIN3-related genes were relevant to actin binding, actin filament binding, cell cortex part and so on (Fig. **4D**). The KEGG analysis results indicated that BIN3 might affect ESCA tumorigenesis by endocytosis (Fig. **4E**).

### 3.5 GSEA Analysis of BIN3 in ESCA

**Fig. (5):**
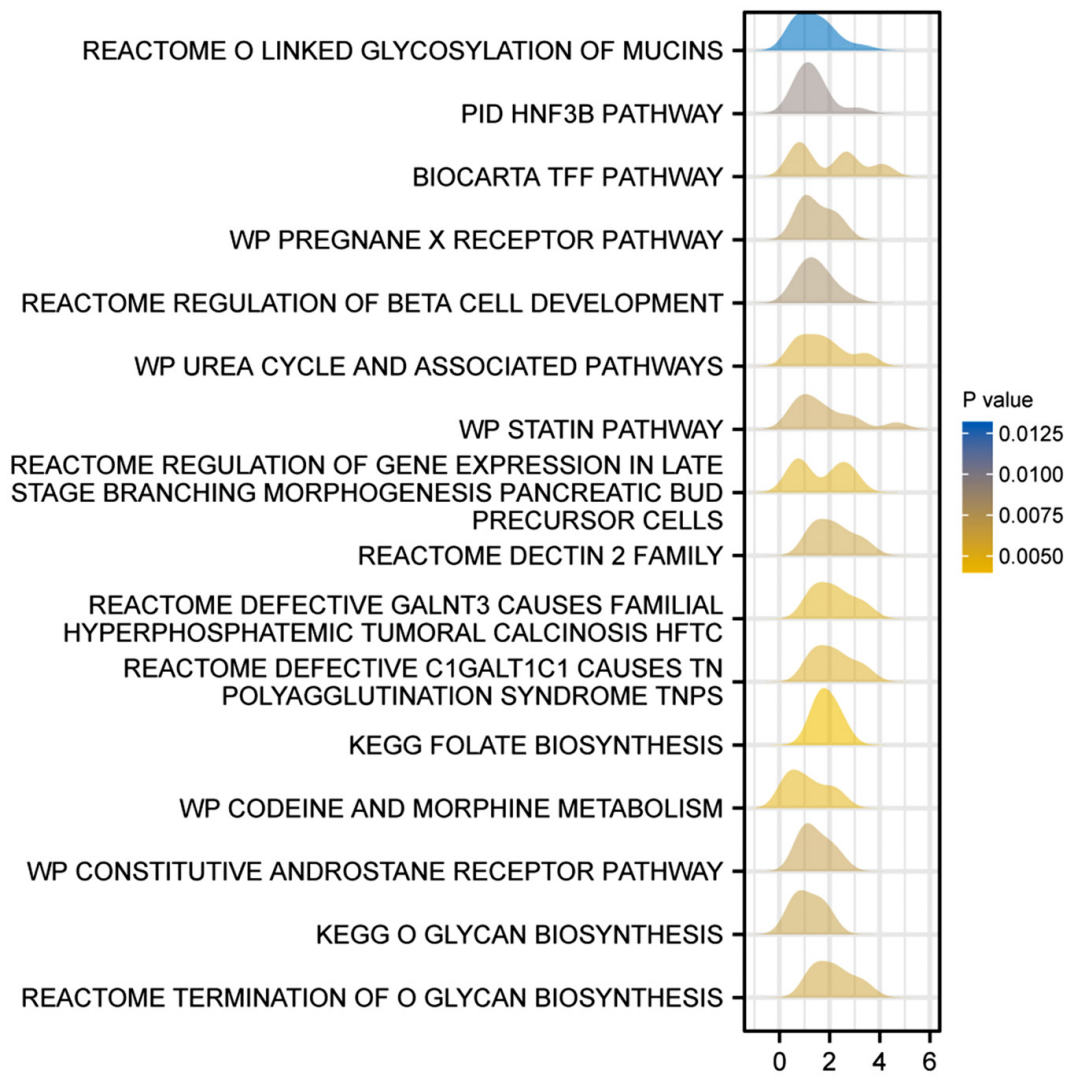
Enrichment plots from gene set enrichment analysis (GSEA).

Additionally, we performed gene set enrichment analysis (GSEA) analysis and observed that BIN3 was associated with certain reactomes and pathways such as O linked glycosylation of mucins, PID HNF3B pathway, biocarta TFF pathway, WP pregnane X receptor pathway, reactome regulation of beta cell development, WP Urea cycle and associated pathways, WP statin pathway and others based on the descending order of the normalized enrichment score (NES) (Fig. **5** and Table **4**).

### 3.6 Immune Infiltrates Correlation of BIN3 in ESCA

**Fig. (6):**
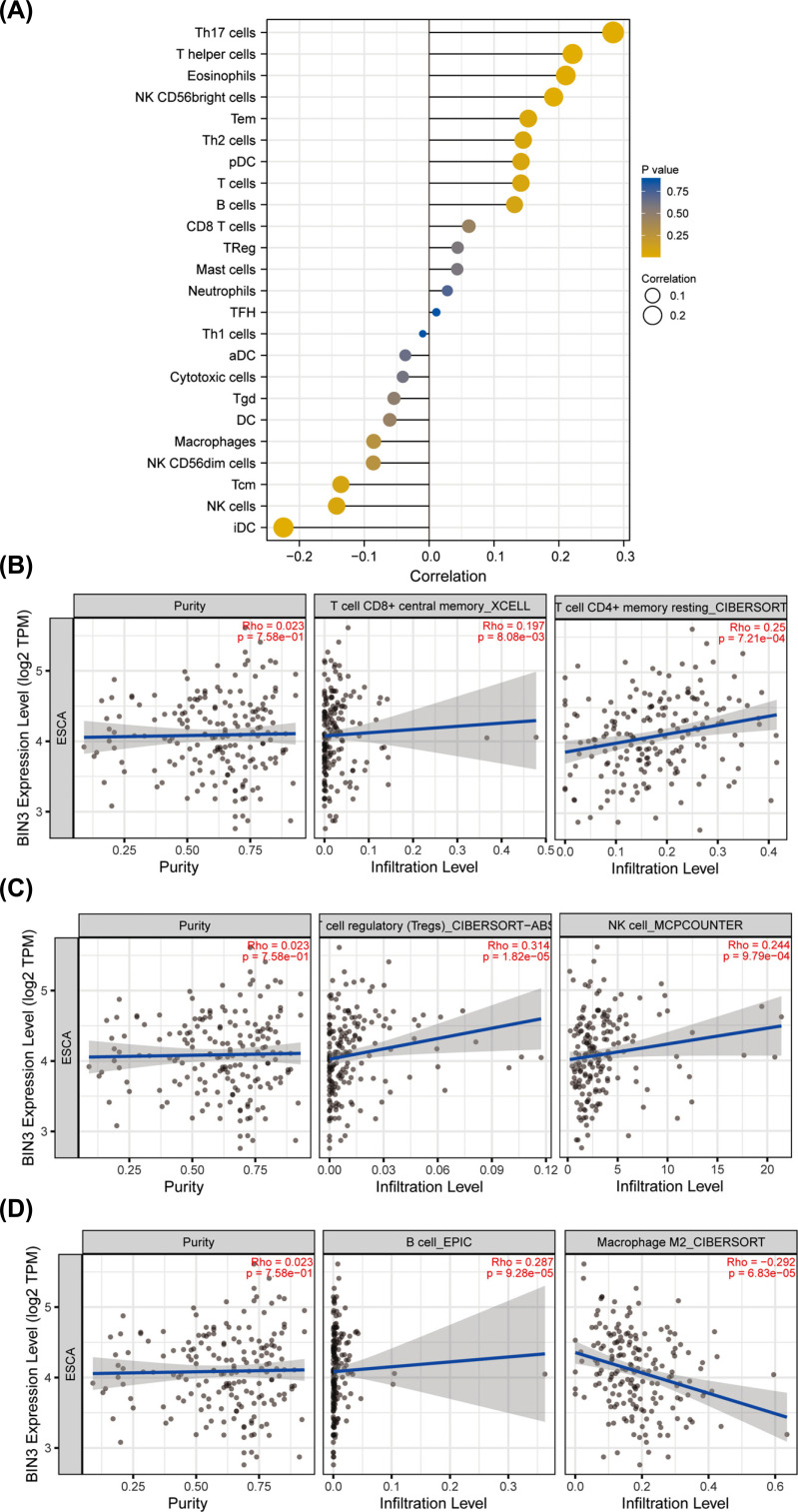
Association analysis of BIN3 gene expression and immune infiltration. (**A**) Correlation analysis between BIN3 expression and immune cells. aDC [activated DC]; B cells; CD8 T cells; Cytotoxic cells; DC; Eosinophils; iDC [immature DC]; Macrophages; Mast cells; Neutrophils; NK CD56bright cells; NK CD56dim cells; NK cells; pDC [Plasmacytoid DC]; T cells; T helper cells; Tcm [T central memory]; Tem [T effector memory]; Tfh [T follicular helper]; Tgd [T gamma delta]; Th1 cells; Th17 cells; Th2 cells; Treg. (B-D) Correlation analysis of BIN3 expression with immune infiltration levels of T cells CD8^+^ and CD4^+^ cells (**B**); Tregs and NK cells (**C**), and B cells and Macrophages M2 cells (**D**).

Tumor-infiltrating lymphocytes are closely related to the tumorigenesis, development and progression of cancers. We performed the correlation assessment between BIN3 expression and the immune infiltration in ESCA using two different algorithms. The lollipop image displayed the relationship between BIN3 and twenty-four immune cells in ESCA and observed that there was a significant relation between BIN3 expression and Th17 cells, T helper cells and immature DC (Fig. **6A**) by ssGSEA algorithm. In addition, we observed that there was a positive relationship between BIN3 and the infiltration level of T cells CD8+ (R = 0.197, *p <* 0.001), T cells CD4^+^ (R = 0.25, *p <* 0.001), Tregs (R = 0.314, *p <* 0.001), NK cells (R = 0.244, *p <* 0.001) and B cells (R = 0.287, *p <* 0.001) (Figs. **6B-6D**). There was a negative relationship between BIN3 expression and the infiltration level of macrophage M2 (R = -0.292, *p <* 0.001, Fig. **6D**).

### 3.7 Preliminary Function Verification of BIN3 in TE-1 Cells

**Fig. (7):**
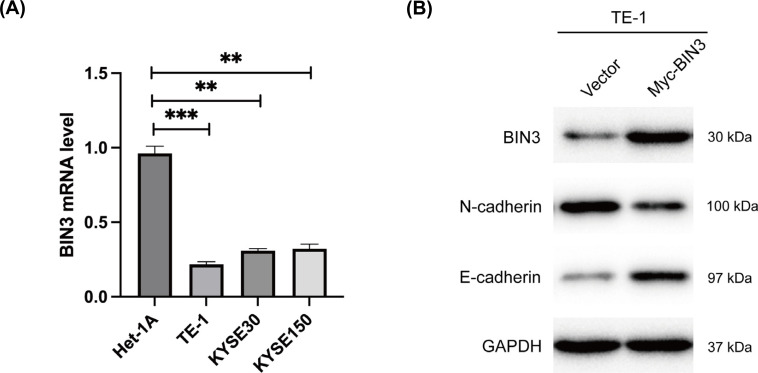
BIN3 expression and the effects on epithelial-mesenchymal transition in ESCA cells. (**A**) RNA level of BIN3 in ESCA cell lines was detected by qRT-PCR. (**B**) EMT related protein levels affected by BIN3 were examined by western blot.

To verify the role of BIN3 in ESCA, we performed gain-of-function experiments *in vitro*. Consistent with the bioinformatics analysis in ESCA tumor tissues, BIN3 mRNA level was significantly decreased in ESCA cell lines (TE-1, KYSE30 and KYSE150) compared to normal human esophagus epithelial cell line Het-1A (Fig. **7**). What’s more, BIN3 upregulation led to the decreased expression of N-cadherin while the increased expression of E-cadherin in TE-1 cells, suggesting that BIN3 might suppress epithelial-mesenchymal transition (EMT) in ESCA.

## 4 DISCUSSION

Our analysis of the current research advances our understanding of the role and function of BIN3 in ESCA. This work was the first study to generally analyze the relationship between BIN3 expression and the clinical values of patients with ESCA such as the clinical characteristics and the prognostic significance. It was screened and validated that the expression of BIN3 was correlated with OS, DSS and PFI survival of ESCA patients, as well as certain clinical features. Tumor immune infiltration analysis indicated that there was a strong correlation between levels of immune cell infiltration and the expression of BIN3. Western blot was performed to initially validate the role of BIN3 in ESCA *in vitro*. Based on the bioinformatics data and experiments, our findings suggest that BIN3 may act as a tumor-suppressor role in ESCA and might be a potential prognostic factor for ESCA patients. In the future, BIN3 combined with magnetic nanoparticles coated with noble metals might be applied for a potential theragnosis [20].

BIN1 and BIN3 are known to belong to the members of the BAR adapter gene family, which are well evolutionarily conserved from yeast to humans [21]. Multiple studies have shown that BIN1 suppressed several cancers' development and progression. For example, it was reported that the ablation of BIN1 could accelerate the susceptibility to cancer especially lung cancer during aging in humans [22]. A previous report showed that loss of BIN1 led to nodal metastasis and decreased outcomes for breast cancer patients [23]. Another study indicated that BIN1 overexpression could restrain esophageal squamous cell carcinoma tumorigenesis [24]. Despite both BIN1 and BIN3 ubiquitously expressed in human tissues. Little was known about the role of BIN3 in tumorigenesis. It was previously reported that low BIN3 expression was an independent predictor of unfavorable survival in patients with primary colorectal cancer [25] Thus, it was supposed that BIN3 might also be a tumor inhibitor. Results from our study showed that BIN3 and AXIN1 were both significantly associated with better OS, DSS and PFI prognosis for ESCA patients. However, only BIN3 was downregulated in ESCA tumor tissues compared to normal tissues, suggesting a tumor suppressor role in tumorigenesis. Moreover, decreased BIN3 expression was associated with certain clinical features such as advanced stages of ESCA. These data indicate that BIN3 was a tumor inhibitor in ESCA.

We also found that BIN3 was closely related to the tumor immune microenvironment by different algorithms. Notably, it was the first study to link BIN3 expression with T cells immune infiltration, as well as the prognosis analysis. Firstly, we assessed the relationship between BIN3 and immune cell populations using the ssGSEA algorithm. There was an obvious correlation between BIN3 and Th 17 cells, T helper cells, Eosinophils and NK cells. Next, we evaluated the association between BIN3 expression and tumor immune infiltrate levels using TIMER2 databases. We found that there was a positive association of BIN3 expression with T cells CD8^+^, T cells CD4^+^, Tregs, B cells and NK cells while a negative relationship between BIN3 expression and macrophage M2. It is known that most T and B cells could have a positive impact on the prognosis of patients with tumors. Moreover, increasing studies have shown that tumor-infiltrating M2 macrophages are closely associated with esophageal cancer tumorigenesis [26, 27]. For example, it was reported that FOXO1 could facilitate esophageal squamous cell carcinoma (ESCC) tumor progression by promoting M2 macrophage infiltration [28]. In ESCC animal studies, the inhibition of CCL2-CCR2 axis could suppress tumor growth by blocking M2 macrophage recruitment and thus enhance the antitumor effect of CD8_+_ T cells [29]. A study showed that HPV16 could accelerate cell migration and invasion of ESCC by recruiting tumor-associated macrophages [30]. These findings suggest that the tumor microenvironment plays a key role in tumor development. Our results reveal that BIN3 is strongly associated with immune infiltration and may influence tumorigenesis of ESCA.

Besides, our experiments preeminently showed that BIN3 overexpression could promote ESCA EMT *in vitro*, which was consistent with our bioinformatics analysis. However, there were some limitations in this research. First, it is better to verify the role of BIN3 by using much more datasets. Second, BIN3 expression at the protein level should be further validated by collecting ESCA samples. Third, the tumor suppressor role of BIN3 in ESCA should be investigated by much more *in vitro* experiments and *in vivo* studies.

## CONCLUSION

In summary, low expression of BIN3 is related to a higher stage and poor prognosis of ESCA. BIN3 expression is positively related to the immune infiltrating levels of T cells, Tregs cells, B cells and NK cells, and a negative correlation with macrophage M2 cells. BIN3 is involved in certain critical pathways in ESCA. Additionally, BIN3 overexpression could promote E-cadherin expression and inhibit N-cadherin expression. Above all, our results suggest that BIN3 may play a tumor-suppressor role in ESCA and serve as a potential biomarker in the prognosis of ESCA.

## AUTHORS’ CONTRIBUTIONS

DL designed the project. WD, GH and XX collected the data, performed the statistical analysis and wrote the manuscript. DL revised the manuscript. All authors read and approved the final manuscript.

## Figures and Tables

**Fig. (1):**
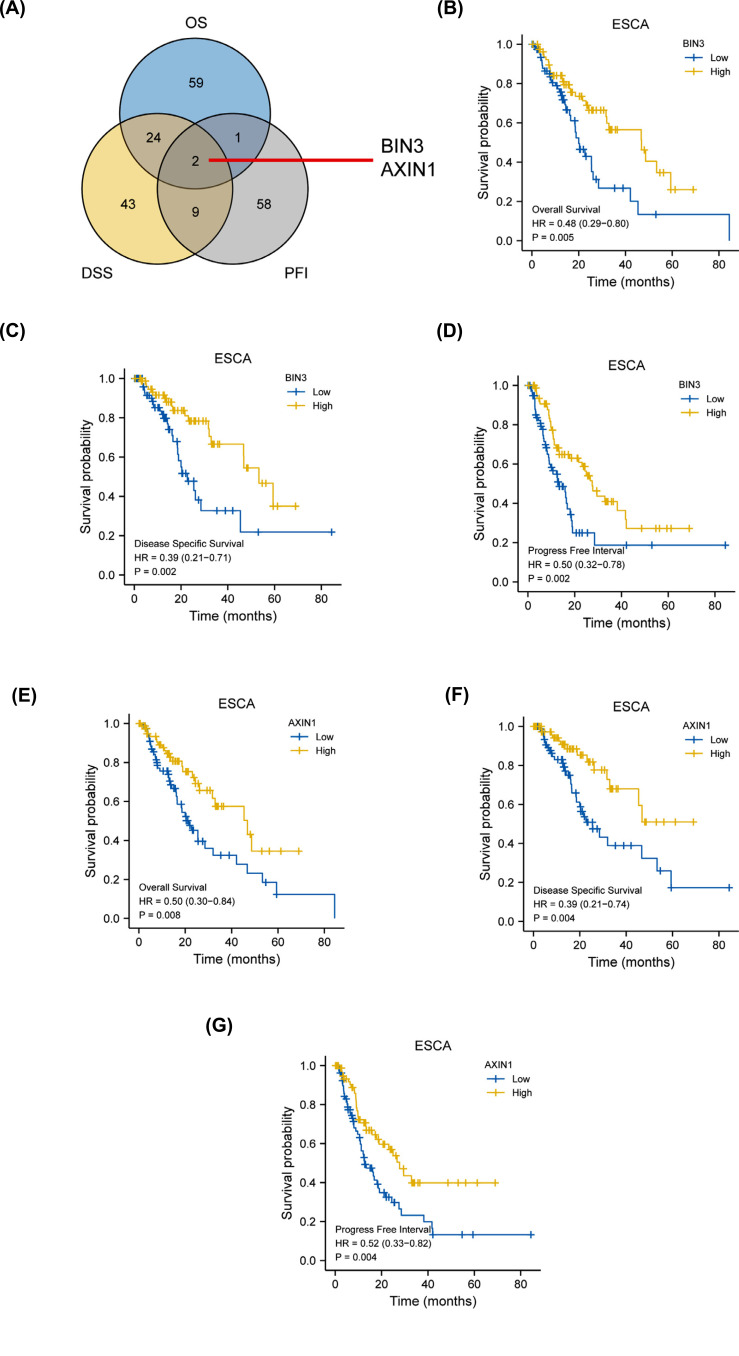
Overlap of prognosis-related genes in ESCA. (**A**) The intersection of OS, DSS, PFI related genes is shown; (**B**) OS curve of BIN3 (**C**) DDS curve of BIN3; (**D**) PFI curve of BIN3; (**E**) OS curve of AXIN1; (**F**) DDS curve of AXIN1; (**G**) PFI curve of BIN3. OS: overall survival. DSS: disease-specific survival. PFI: progression free interval.

**Fig. (3):**
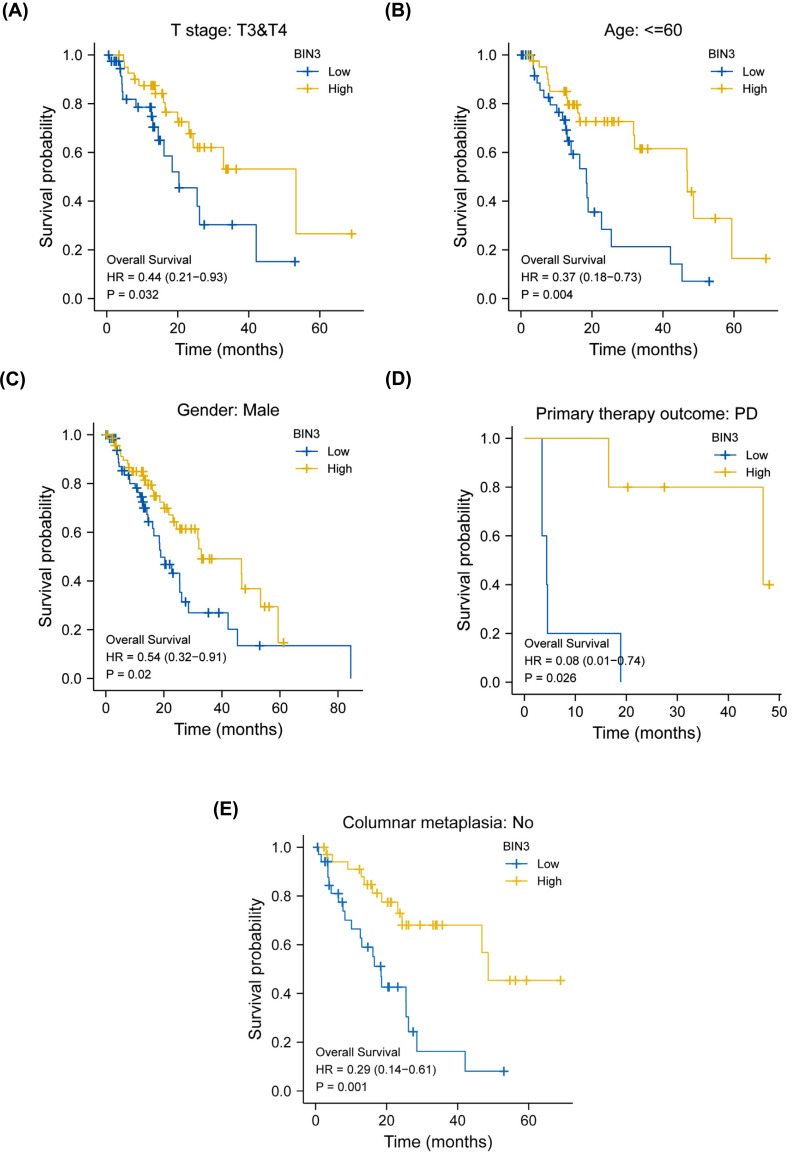
Overall survival analysis for ESCA patients with BIN3 expression levels. (**A**) High expression of BIN3 is associated with better survival of patients with T stage 3 and T stage 4 (*p =* 0.032). (**B**) High expression of BIN3 is associated with better survival of patients under the age of sixty (*p* = 0.004). (**C**) High expression of BIN3 is associated with better survival of ESCA male patients (*p* = 0.02). (**D**) High expression of BIN3 is associated with better survival of ESCA patients with primary therapy outcome (*p* = 0.026). (**E**) High expression of BIN3 is associated with better survival of ESCA patients with no columnar metaplasia (*p* = 0.001).

**Table 1:** Association of BIN3 expression and clinicopathological features in ESCA.

**Characteristic**	**Low Expression of BIN3**	**High Expression of BIN3**	***p***	**Statistic**
n	81	81	-	-
T stage, n (%)	-	-	0.033	-
T1	7 (4.8%)	20 (13.8%)	-	-
T2	21 (14.5%)	16 (11%)	-	-
T3	41 (28.3%)	36 (24.8%)	-	-
T4	3 (2.1%)	1 (0.7%)	-	-
N stage, n (%)	-	-	0.074	-
N0	25 (17.4%)	41 (28.5%)	-	-
N1	37 (25.7%)	26 (18.1%)	-	-
N2	6 (4.2%)	3 (2.1%)	-	-
N3	3 (2.1%)	3 (2.1%)	-	-
M stage, n (%)	-	-	0.486	-
M0	63 (48.8%)	58 (45%)	-	-
M1	3 (2.3%)	5 (3.9%)	-	-
Pathologic stage, n (%)	-	-	-	-
Stage I	3 (2.1%)	13 (9.2%)	-	-
Stage II	33 (23.2%)	36 (25.4%)	-	-
Stage III	32 (22.5%)	17 (12%)	-	-
Stage IV	3 (2.1%)	5 (3.5%)	-	-
OS event, n (%)	-	-	0.336	0.92
Alive	45 (27.8%)	52 (32.1%)	-	-
Dead	36 (22.2%)	29 (17.9%)	-	-
Gender, n (%)	-	-	0.072	3.24
Female	7 (4.3%)	16 (9.9%)	-	-
Male	74 (45.7%)	65 (40.1%)	-	-
Race, n (%)	-	-	< 0.001	
Asian	28 (19.4%)	10 (6.9%)	-	-
Black or African American	4 (2.8%)	2 (1.4%)	-	-
White	40 (27.8%)	60 (41.7%)	-	-
Age, n (%)	-	-	0.116	2.47
<=60	47 (29%)	36 (22.2%)	-	-
>60	34 (21%)	45 (27.8%)	-	-
Weight, n (%)	-	-	0.003	9.05
<=70	48 (30%)	28 (17.5%)	-	-
>70	32 (20%)	52 (32.5%)	-	-
Height, n (%)	-	-	0.589	0.29
< 170	26 (17%)	21 (13.7%)	-	-
>=170	52 (34%)	54 (35.3%)	-	-
BMI, n (%)	-	-	0.002	9.89
<=25	53 (34.6%)	31 (20.3%)	-	-
>25	25 (16.3%)	44 (28.8%)	-	-
Histological type, n (%)	-	-	< 0.001	20.77
Adenocarcinoma	25 (15.4%)	55 (34%)	-	-
Squamous cell carcinoma	56 (34.6%)	26 (16%)	-	-
Histologic grade, n (%)	-	-	0.874	0.27
G1	8 (6.3%)	8 (6.3%)	-	-
G2	35 (27.8%)	31 (24.6%)	-	-
G3	25 (19.8%)	19 (15.1%)	-	-
Smoker, n (%)	-	-	0.811	0.06
No	25 (17.4%)	22 (15.3%)	-	-
Yes	48 (33.3%)	49 (34%)	-	-
Age, median (IQR)	59 (53, 67)	63 (54, 74)	0.073	2745.5

**Table 2:** Logistic regression analysis of BIN3 expression associated with clinical pathological characteristics.

**Characteristics**	**Total (N)**	**Odds Ratio (OR)**	***P*-value**
T stage (T3&T4 *vs*. T1&T2)	145	0.654 (0.336-1.262)	0.207
N stage (N1&N2&N3 *vs*. N0)	144	0.424 (0.215-0.825)	0.012
M stage (M1 *vs*. M0)	129	1.810 (0.425-9.141)	0.430
Pathologic stage (Stage III&Stage IV *vs*. Stage I&Stage II)	142	0.462 (0.230-0.910)	0.027
Tumor cental location (Mid&Proximal *vs*. Distal)	161	0.420 (0.205-0.836)	0.015
Barretts esophagus (Yes *vs*. No)	132	1.600 (0.674-3.955)	0.294
Columnar metaplasia (Yes *vs*. No)	98	1.545 (0.639-3.851)	0.339
Columnar mucosa dysplasia (High grade dysplasia *vs*. Negative/no dysplasia & Low grade dysplasia)	68	5.556 (1.859-19.277)	0.004

**Table 3:** Univariate and multivariate Cox proportional hazards analysis for BIN3 expression.

**Characteristics**	**Total** **(N)**	**Univariate Analysis**		**Multivariate Analysis**	
		**HR (95% CI)**	***P*-value**	**HR (95% CI)**	***P*-value**
Age	162	-	-	-	-
<=60	83	Reference	-	-	
>60	79	0.831 (0.506-1.365)	0.466	1.109 (0.872-3.185)	0.267
T stage	145	-	-	-	-
T1&T2	64	Reference	-	-	-
T3&T4	81	1.312 (0.756-2.277)	0.334	1.879 (1.625-4.312)	0.238
N stage	144	-	-	-	-
N0&N1	129	Reference	-	-	-
N2&N3	15	1.944 (0.904-4.181)	0.089	1.353 (0.511-3.580)	0.543
M stage	129	-	-	-	-
M0	121	Reference	-	-	-
M1	8	5.075 (2.312-11.136)	<0.001	2.869 (1.176-7.002)	0.021
Pathologic stage	142	-	-	-	-
Stage I & Stage II	85	Reference	-	-	-
Stage III & Stage IV	57	3.223 (1.807-5.747)	<0.001	2.831 (1.362-5.883)	0.005
Histological type	162	-	-	-	-
Adenocarcinoma	80	Reference	-	-	-
Squamous cell carcinoma	82	0.875 (0.526-1.455)	0.607	1.325 (0.817-3.825)	0.416
Tumor central location	161	-	-	-	-
Distal	113	Reference	-	-	-
Mid & Proximal	48	0.952 (0.502-1.805)	0.880	1.224 (0.886-3.825)	0.321
Columnar mucosa dysplasia	68	-	-	-	-
Negative/no dysplasia& Low grade dysplasia	43	Reference	-	-	-
High grade dysplasia	25	0.884 (0.446-1.749)	0.722	1.675 (0.865-4.452)	0.617
Columnar metaplasia	98	-	-	-	-
No	70	Reference	-	-	-
Yes	28	1.133 (0.628-2.046)	0.678	1.092 (0.873-1.967)	0.156

**Table 4:** Results of gene set enrichment analysis for BIN3.

**Description**	**setSize**	**Enrichment Score**	**NES**	***p* value**	***p*.adjust**
REACTOME_O_LINKED_GLYCOSYLATION_OF_MUCINS	62	0.625513548	2.529980727	0.013157895	0.028339307
PID_HNF3B_PATHWAY	45	0.606144165	2.331360418	0.009433962	0.026215738
BIOCARTA_TFF_PATHWAY	24	0.728269485	2.313762751	0.00617284	0.026215738
WP_PREGNANE_X_RECEPTOR_PATHWAY	33	0.655161799	2.300653488	0.007407407	0.026215738
REACTOME_REGULATION_OF_BETA_CELL_DEVELOPMENT	42	0.597046173	2.289347201	0.008333333	0.026215738
WP_UREA_CYCLE_AND_ASSOCIATED_PATHWAYS	21	0.725900728	2.277722781	0.005376344	0.026215738
WP_STATIN_PATHWAY	31	0.645292983	2.244311897	0.006666667	0.026215738
REACTOME_REGULATION_OF_GENE_EXPRESSION_IN_LATE_STAGE_BRANCHING_MORPHOGENESIS_PANCREATIC_BUD_PRECURSOR_CELLS	16	0.752058201	2.205484613	0.004807692	0.026215738
REACTOME_DECTIN_2_FAMILY	26	0.672965684	2.203071836	0.00621118	0.026215738
REACTOME_DEFECTIVE_GALNT3_CAUSES_FAMILIAL_HYPERPHOSPHATEMIC_TUMORAL_CALCINOSIS_HFTC_	16	0.749004771	2.196530127	0.004807692	0.026215738
REACTOME_DEFECTIVE_C1GALT1C1_CAUSES_TN_POLYAGGLUTINATION_SYNDROME_TNPS_	17	0.740885856	2.191187369	0.005128205	0.026215738
KEGG_FOLATE_BIOSYNTHESIS	11	0.829482216	2.171713292	0.004016064	0.026215738
WP_CODEINE_AND_MORPHINE_METABOLISM	15	0.755372017	2.171602492	0.004608295	0.026215738
WP_CONSTITUTIVE_ANDROSTANE_RECEPTOR_PATHWAY	32	0.603851777	2.118857497	0.006666667	0.026215738
KEGG_O_GLYCAN_BIOSYNTHESIS	30	0.606067825	2.096784658	0.006451613	0.026215738
REACTOME_TERMINATION_OF_O_GLYCAN_BIOSYNTHESIS	23	0.664484482	2.08532699	0.006024096	0.026215738
KEGG_MATURITY_ONSET_DIABETES_OF_THE_YOUNG	25	0.648740813	2.057649287	0.006410256	0.026215738
BIOCARTA_MITOCHONDRIA_PATHWAY	19	0.68537917	2.055665141	0.005434783	0.026215738
REACTOME_DISEASES_ASSOCIATED_WITH_O_GLYCOSYLATION_OF_PROTEINS	67	0.502434326	2.054904911	0.014084507	0.028339307
REACTOME_SYNTHESIS_OF_PA	39	0.544643352	2.0340133	0.007692308	0.026215738

## Data Availability

The authors declare that the data and materials of this study are available within the article.

## LIST OF ABBREVIATIONS

BIN3: Bridging Integrator 3
ESCA: Esophagus Carcinoma
DSS: Disease-Specific Survival
PFI: Progression-Free Internal
TCGA: The Cancer Genome Atlas
GSEA: Gene Set Enrichment Analysis
OS: Overall Survival
DSS: Disease-Specific Survival
PFI: Progression-Free Interval
EMT: Epithelial-Mesenchymal Transition
