# Supplementary material for: Bridging Integrator 3 (BIN3) Downregulation Predicts a Poor Prognosis in Patients with Esophagus Carcinoma: A Study based on TCGA Data
Source: Comb Chem High Throughput Screen. 2023 Jun 12;26(11):1974–89. doi: 10.2174/1386207326666221205101815 (PMC10332122; doi:10.2174/1386207326666221205101815)
Supplement: Supplementary file 1 [file CCHTS-26-1974_SD1.zip › CCHTS-26-1974_SD1/BMS-CCHTS-2022-141 Suppl.-MS.pdf]

Supplementary Material

Bridging Integrator 3 (BIN3) Downregulation Predicts a Poor Prognosis in Patients with Esophagus Carcinoma: A Study Based on TCGA Data

Daohang Li[1,*], Weiming Deng[1], Guozheng Huang[1] and Xin Xiao[1]

[1]*Department of Thoracic Surgery, Chaohu Hospital affiliated with Anhui Medical University, Hefei, 238000, China*

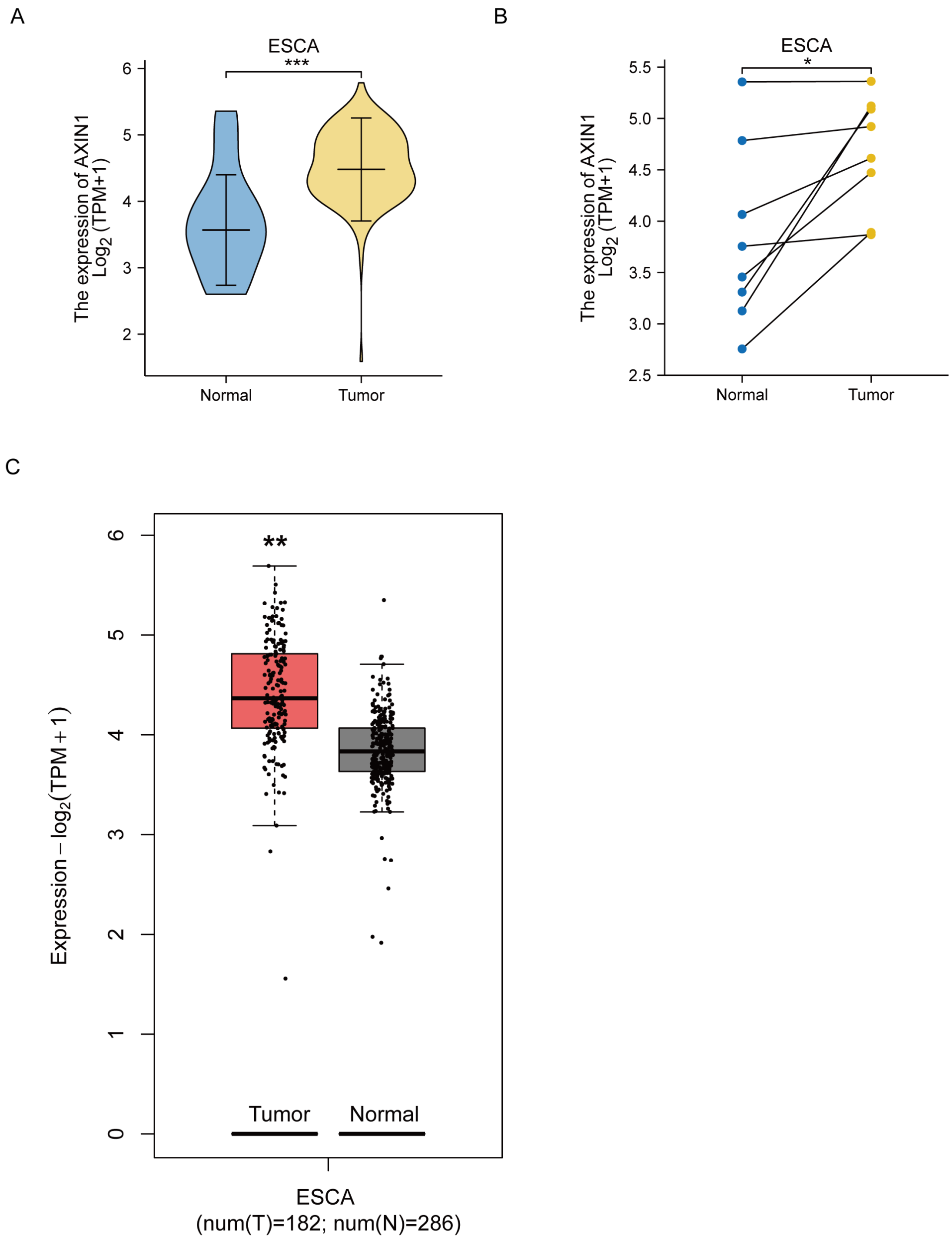

Supp Fig. (1).
